# Efficacy of environmental DNA to detect and quantify stream tadpoles of *Odorrana splendida*

**DOI:** 10.1098/rsos.181798

**Published:** 2019-01-09

**Authors:** Noriko Iwai, Kiyomi Yasumiba, Teruhiko Takahara

**Affiliations:** 1 Department of Environment Conservation, Tokyo University of Agriculture and Technology, 3-5-8 Saiwai-cho, Fuchu, Tokyo 183-0054, Japan; 2 Faculty of Life and Environmental Science, Shimane University, 1060 Nishikawatu-cho Matsue, Shimane 690-8504, Japan

**Keywords:** abundance, Amami-Oshima Island, biomass, Ishikawa's frog, lotic system, quantitative PCR

## Abstract

Environmental DNA (eDNA) can be used to detect and estimate the density of rare or secretive species, especially in aquatic systems. However, the efficacy of eDNA method has not been validated in lotic systems. We examined the efficacy of the eDNA method to detect and estimate abundance and biomass of a stream-dwelling frog species, *Odorrana splendida*. We conducted eight field surveys over 2 years and obtained 53 water samples from 10 streams with known distribution of *O. splendida* tadpoles. The eDNA method accurately detected the presence of *O. splendida* in 79.2% of survey samples. The amount of *O. splendida* eDNA (copies s^−1^) in the water samples fluctuated seasonally and each site showed different peaks during different seasons. The relationship between the abundance or biomass of tadpoles and the amount of eDNA was significantly positive, but was not strong, probably because of a large difference in the relationship patterns among streams. In lotic systems, water flow might prevent even distribution of eDNA and thus make it difficult to obtain eDNA reflecting its total amount in the water. Sampling a larger amount of water or higher number of subsamples might more accurately reflect the presence and absolute amount of eDNA in water.

## 1. Introduction

Understanding population distribution and density is critical to conserve endangered species [1]. However, obtaining such data is often difficult and time-consuming, and sometimes unreliable [2]. Detection of environmental DNA (eDNA; DNA fragments released from organisms into environment media such as soil and water) is a relatively recently developed approach that can increase the accuracy and decrease the cost of data collection [3]. eDNA allows detection of rare or secretive species, especially in aquatic systems [4]. Several studies have reported the utility of eDNA in detecting aquatic species in lentic systems [5–7]. However, studies validating the eDNA method in lotic systems are still limited, and the method is usually only applied to relatively large animals, such as fish [8,9]. Detection of eDNA from organisms may be more difficult in flowing water due to dilution and dispersion by dynamic hydrological processes [9].

Although the exact sources of eDNA are still unknown, they can include urine, faeces, sloughed skin and dead bodies of animals [3]. Higher numbers of target species create more sources of eDNA; therefore, the amount of eDNA in a water sample probably correlates with the number of target animals in the water. Thus, some studies have succeeded in estimating the abundance and/or biomass of target species from the concentration of eDNA in lentic systems. For example, the concentration of eDNA positively correlated with carp biomass in experimental ponds [10], and with the density of tadpoles and newts in natural ponds [6]. However, in lotic systems, the dilution and length of flow from the eDNA source can make the estimation of biomass difficult [11]. There are only a few studies that have validated the efficacy of the eDNA method in estimating the abundance and biomass of target species in lotic systems. These studies have shown variable results; some have shown that the eDNA concentration in flowing water positively correlates with the abundance or biomass of the target species upstream [12,13], whereas, others have shown no relationship [14].

The goal of this study was to examine the efficacy of the eDNA method in detecting and estimating the abundance and biomass of a stream-dwelling species. We targeted stream tadpoles of *Odorrana splendida*, which are endemic to Amami-Oshima Island, Japan. The species is listed as endangered in the International Union for Conservation of Nature red list, owing to habitat degradation and introduced predators [15,16]. To conserve this frog, there is a need to understand the distribution and density of tadpoles across the island. Our aims were to (1) elucidate the detection rate of *O. splendida* using the eDNA method in the field, (2) examine seasonal fluctuations in eDNA in natural streams and (3) examine the relationship between abundance and biomass of tadpoles and the amount of eDNA released into stream water.

## 2. Material and methods

### 2.1. Study species

We targeted tadpoles of *Odorrana splendida*, a forest frog that breeds from February to April [17] and lays eggs in rock crevices or in water flowing under the ground of rocky headstreams [18]. After hatching, tadpoles spend a relatively long larval period, sometimes exceeding 2 years in the stream [19]. They grow up to a snout–vent length (SVL) of 1.85 cm and weigh approximately 1 g (wet weight); they metamorphose from late June to early September [19]. The tadpoles can disperse from the breeding site to around 90 m yr^–1^ [20], and the estimated density around the breeding site has been reported to be 266.5 individuals m^−2^ [21].

### 2.2. Field survey

**Figure 1.**
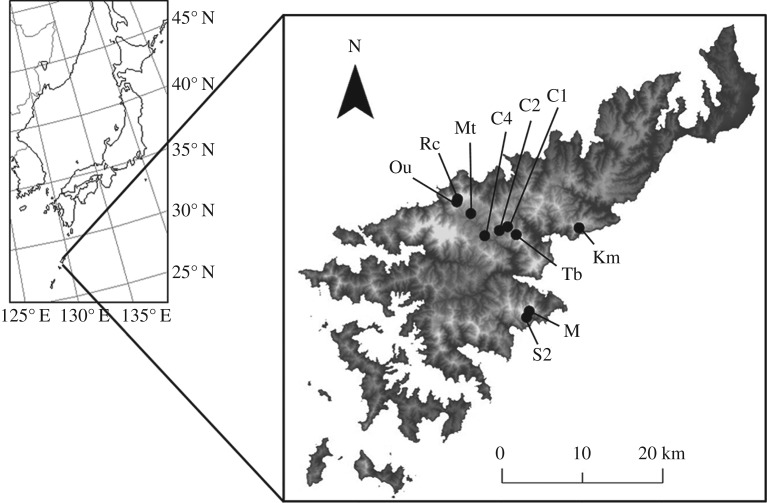
Map of the study area. The dots show the 10 stream sampling sites.

We conducted field surveys at 10 headwater stream sites on Amami-Oshima Island (28.30° N, 129.40° E, figure 1) in 2015 and 2017. Our previous surveys have confirmed the presence of *O. splendida* tadpoles in the 10 streams [22]. The surveys in 2015 were conducted mainly to investigate whether eDNA can detect the presence of the target species. The surveys in 2017 were conducted to investigate the relationship between the abundance and biomass of tadpoles and the amount of eDNA. The surveys were conducted three times in 2015 and five times in 2017. Owing to the constraint of time and loss of access to some sites after heavy rains, not all the sites were surveyed every time. In 2015, we collected water samples from 8, 2 and 7 sites in August, October and December, respectively. In 2017, we collected water samples from 10, 5, 7, 6 and 8 sites in February, May, July, August and November, respectively.

Water samples of 500 ml (in 2015) or 1 l (500 ml × 2 in 2017) were collected using sterilized 500 ml bottles. The bottles were then immediately placed in individual plastic bags, transported with a refrigerant to the field station and stored in a freezer until filtration in the laboratory. To prevent contamination between study sites, water sampling was conducted first of all activities at each site. In 2017, we carried 1 l of bottled water that was sourced off the island (i.e. *O. splendida*-DNA free) in two clean 500 ml bottles to the sampling sites as a negative control for the sampling stage (cooler blanks, [12]). The cooler blanks were treated like the other water samples, except that they were not opened at the sampling sites. To account for the difference in discharge, the velocity of water was measured using a current meter (VR-301; KENEK) for subsections at each site and the flow volume was calculated by the sum of discharge from a subsection, which was obtained using the formula: depth × width × velocity.

The presence (in 2015) and the number and size (in 2017) of tadpoles were recorded after water sampling. Tadpoles were visually inspected within 10 m upstream of the water sampling site. In 2017, tadpoles found within 10 m upstream from the sample site were captured using a net and photographed with a scale to indicate size (we counted, but did not capture tadpoles in February due to time constraint). We measured the SVL of each tadpole using the scale and pixels on each photograph and ImageJ software (NIH, USA) and calculated the body size in millimetre. The total biomass of tadpoles was estimated from the equation obtained from measuring 468 *O. splendida* tadpoles in another study (N Iwai 2015, unpublished data): W (g) = 0.206 × SVL (cm)^2.85^.

### 2.3. Real-time quantitative PCR

Water samples were vacuum-filtered through glass microfibre filters (GF/F; GE Healthcare Bio-Sciences, Pittsburgh, PA, USA) with 0.7 μm particle retention. Sodium hypochlorite treatment (10% for 5 min, rinsed with distilled water) was performed in between samples to avoid contamination [23]. Filtering control (i.e. 500 ml of distilled water) was filtered as a last sample on each day of sample filtration in 2015. The filter papers were wrapped in aluminium foil, individually placed in plastic bags to prevent contamination, and stored at −30°C. The DNA was extracted from the residue according to the method of Uchii *et al*. [24], using a Salivette tube (Sarstedt, Nümbrecht, Germany) and a DNeasy Blood and Tissue Kit for DNA purification (Qiagen, Hilden, Germany) in a reaction mixture of final volume 100 µl. In brief, the residue was incubated by submersion in a mixed buffer (400 µl of buffer AL and 40 µl of Proteinase K; Qiagen) using a Salivette tube in a dry oven at 56°C for 30 min. The tubes were centrifuged at 5000*g* for 5 min at 25°C. Subsequently, the supernatant was removed and 220 µl of TE (Tris-EDTA) buffer (pH: 8.0; 10 mM Tris–HCl and 1 mM EDTA) was added to the pellet and the tubes were centrifuged again at 5000*g* for 5 min. Buffer AL (200 µl) and 100% ethanol (600 µl) were then added to each tube and mixed by pipetting. The mixture was applied to a DNeasy Mini spin column and centrifuged at 6000*g* for 1 min. This step was repeated until the mixture was completely processed. We followed the manufacturer's instructions for further steps, and eDNA was eluted from each sample solution with a final volume of 100 µl buffer AE.

Environmental DNA was quantified using a StepOnePlus™ Real-Time PCR system (Life Technologies, Carlsbad, CA, USA). Each TaqMan reaction contained 10 µl of TaqMan^®^ Environmental Master Mix 2.0 (Thermo Fisher Scientific Inc.), 1 µl of *O. splendida* primer (900 nM)/probe (125 nM) mix, 7 µl of distilled water and 2 µl of eDNA extract. We used a primer/probe for *O. splendida* developed by Takahara *et al*. [25]—OsplND5-F, 5′- GCTGAGAAGGCGTTGGAATAA-3′; OsplND5-R, 5′- TGTAGAGGACGGCTTGTAATGC-3′; and OsplND5-Pr, 5'-[FAM]- CCGAAGCGACGCTGCCACC-[TAMRA]-3′. To prove that the primer did not amplify other sympatric frog species, we amplified the DNA extracted from the tissue of *Babina subaspera* and *O. amamiensis*, and confirmed no amplification [25].

The PCR conditions were as follows: 2 min at 50°C, 10 min at 95°C, 55 cycles of 15 s at 95°C, and 60 s at 60°C [10]. To produce standard DNA for the qPCR, a target amplicon was inserted into the pMD20-T vector (Takara Bio, Shiga, Japan), and the vector was digested with BamHI. A dilution series of the plasmid containing 1 × 10^1^ to 1 × 10^4^ copies was amplified as the standards in duplicates in all the qPCR assays. The qPCR for each sample was performed in eight wells, and the mean was used as the concentration of eDNA (copies l^−1^). If any of the eight replicates per sample yielded a positive result, the sample was designated as containing *O. splendida* eDNA. As a negative control, each qPCR assay included eight wells that contained no template (2 µl of DNA-free water; i.e. NTC). The range of qPCR efficiency throughout the study, calculated from the slope of standard curves, was 86.7%–112.9% and the range of standard curve *R*^2^-value was 0.971–1.000.

To avoid contamination, we followed a unidirectional laboratory flow. That is, we performed all PCR protocols, including preparation/addition of the standards, and qPCR cycling in two separate rooms (rooms 1 and 2, respectively). To prevent carry-over contamination, no equipment or samples were moved from room 2 to room 1.

### 2.4. Statistical analyses

As the presence of *O. splendida* was confirmed in all the streams, all the samples were expected to show positive results. Therefore, the detection rate in this study was calculated as the number of samples in which the method detected the presence of the target species out of all the samples (*N* = 53). The amount of eDNA flowing in the stream per second was calculated by multiplying eDNA concentration (the number of eDNA copies per litre) with the flow volume at each site (l s^−1^). The relationships between the abundance and biomass of tadpoles and eDNA amount (copies s^−1^) were analysed using type II regression model with the standard major axis method for all the sites combined. The amount of eDNA was log transformed following the method published previously [6,9]. When the eDNA amount was zero, 1.0 × 10^−5^ was added before log transformation. We used the software package R (v. 3.2.3, [26]) for all statistical analyses.

## 3. Results

Positive results were not obtained for any of the control samples (i.e. cooler blanks, filtering controls and NTC); therefore, contamination was unlikely. We observed more than one *O. splendida* tadpole in 49 of 53 samples, indicating visual survey had 92.5% detection rate (table 1). Environmental DNA of *O. splendida* was detected in 42 samples, indicating that this method detected the presence of *O. splendida* in 79.2% of samples (table 1).

**Table 1.** Detection results by the eDNA method in 53 water samples from 10 streams with known distribution of *Odorrana splendida*. The ratio shows the number amplified out of eight replications in the qPCR assay. Negative results (i.e. 0/8) are shown in italics. We observed tadpoles within 10 m upstream of the sampling site except in four sites (shown as shaded cells). A datum (well) pertaining to stream C4 in Aug 2017 was eliminated due to the exceptional amplification curves during the qPCR assay.

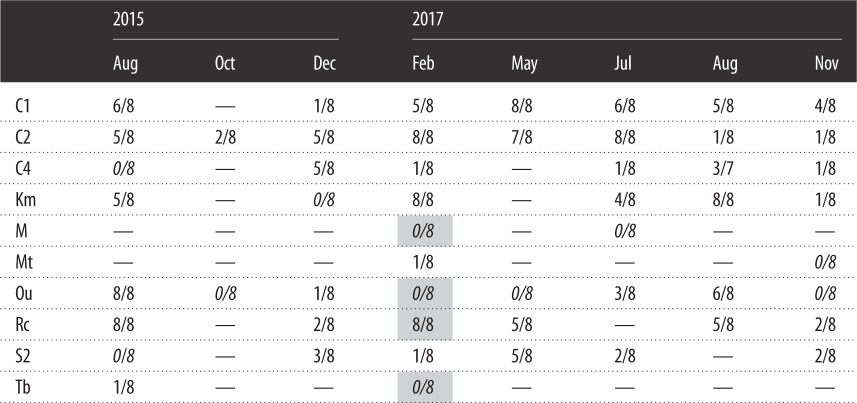

**Figure 2.**
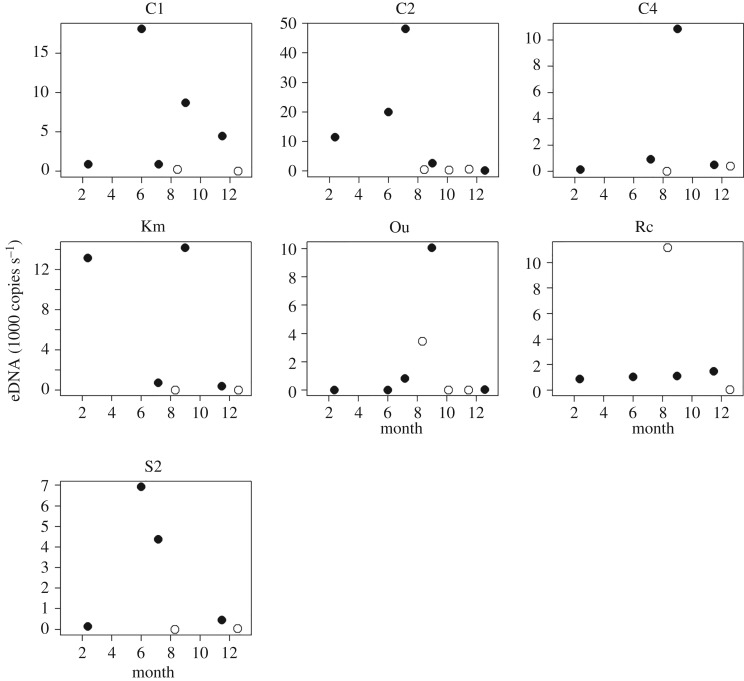
Seasonal fluctuations in the amount of eDNA of *Odorrana splendida* (1000 copies s^−1^) at seven stream sites. Open circles show the data from 2015 and filled circles show data from 2017. The circles were allocated according to the date (not month) of sampling on *x*-axis.

The amount of eDNA (copies s^−1^) in streams showed seasonal fluctuations (figure 2), which differed among seven stream sites. The highest amount of eDNA was detected at two sites in May, one site in July and four sites in August.

**Figure 3.**
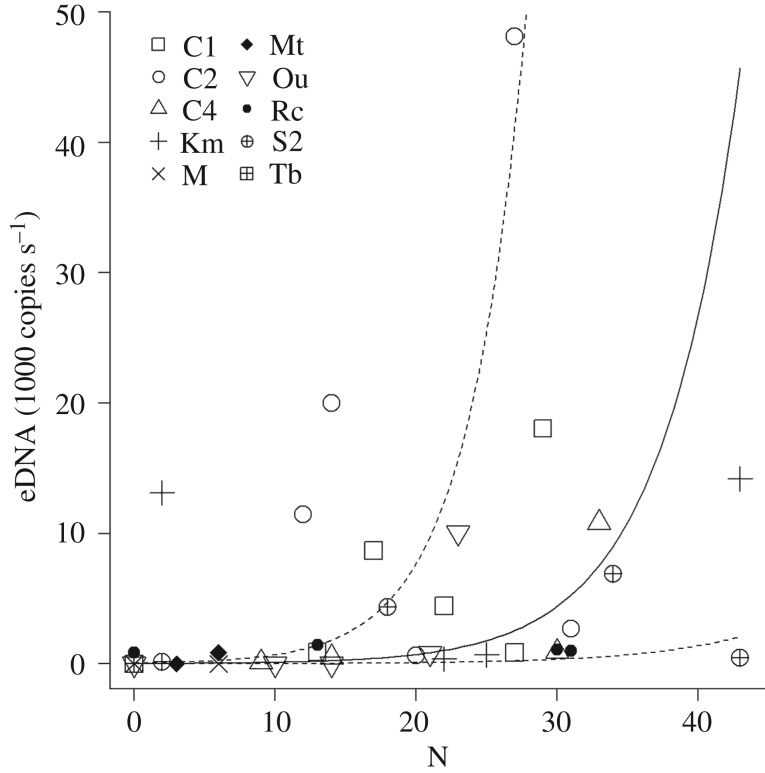
Relationship between the abundance of *Odorrana splendida* tadpoles within 10 m upstream of the sampling site and the amount of eDNA (1000 copies s^−1^). Significant regression line is shown as a solid line with upper and lower limits of the 95% CI for the slope and elevation of the regression shown by dotted lines.

**Figure 4.**
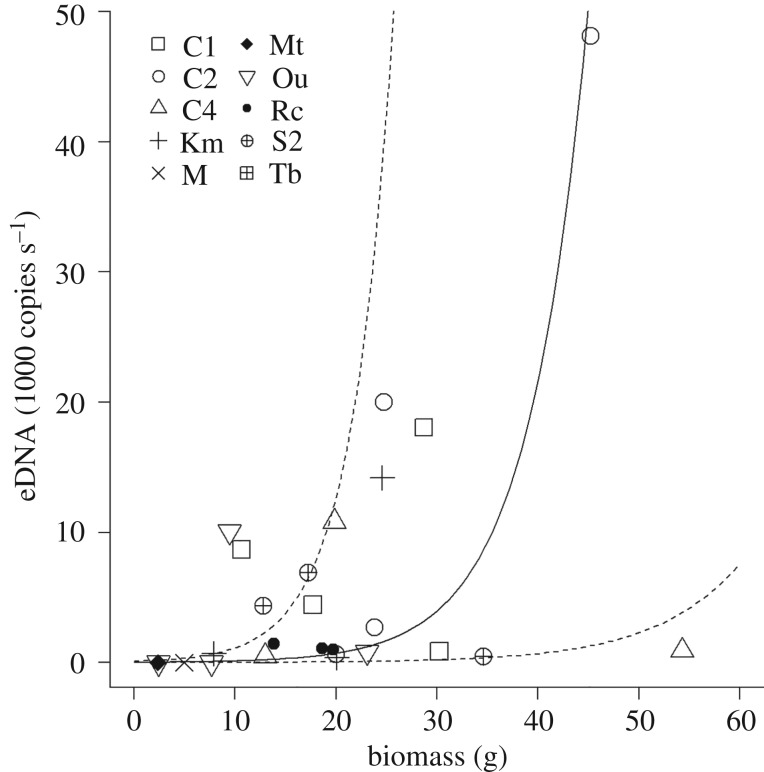
The relationship between the total estimated biomass of *Odorrana splendida* tadpoles within 10 m upstream of each sampling site and the amount of eDNA (1000 copies s^−1^). Significant regression line is shown as a solid line with upper and lower limits of the 95% CI for the slope and elevation of the regression is shown by dotted lines.

In 2017, we found up to 43 tadpoles per site, and the estimated biomass was 54.3 g per site within 10 m upstream of the sampling site, and detected up to 48 133 copies s^−1^ eDNA per site (figures 3 and 4). The relationship between the abundance or biomass of tadpoles and amount of eDNA seemed to differ among streams, but it was significantly positive when the results of all the streams were combined (*r*^2^ = 0.32, *p* < 0.001, figure 3 for abundance; *r*^2^ = 0.29, *p* = 0.005, figure 4, for biomass).

## 4. Discussion

We examined the efficacy of the eDNA method to detect the presence of target species in a lotic system. The detection rate by the eDNA method was 79.2%. The reported detection rate of eDNA varies among studies. In lentic systems, it is from 99.3% to 100% (99.3% in great crested newts [27], and 100% in bluegill [5] and bullfrogs [7]). In lotic systems, it is from 77% to 83.3% (77% in Chinook salmon [28], 83% in brook trout [9] and 83.3% in Japanese clawed salamander [29]). Our study supports the fact that the eDNA method can be used to detect target animals in lotic systems with approximately 80% detection rate, which is lower than that in lentic systems.

In the present study, 22.6% of the samples presented negative eDNA results. Some water samples were eDNA negative even when we observed tadpoles within 10 m upstream of the sampling site. For example, at stream Ou in October 2015, we counted 30 individuals of *O. splendida* in the same pool where we collected water samples, but did not detect eDNA. The reason for this negative result may be because we did not catch floating eDNA in stream water or we collected existing eDNA but it failed to amplify in the PCR assay. The validity of the method used in this study to detect eDNA in water samples has been proved in other studies [10]. In addition, in the supplementary analysis, to test for inhibition in the eDNA samples, we performed a spike experiment using the number of known target DNA copies (1.0 × 10^3^ copies) with a plasmid containing the cytochrome *b* gene of *Hypomesus nipponensis* (a fish not inhabiting our study area) and the primer–probe set from Takahara *et al*. (T Takahara 2018, unpublished data). As a result, the *Δ*Ct values from the internal controls of the four samples with negative eDNA results for qPCR assays were less than 0.20, indicating that they were lower than the inhibition criteria according to Hartman *et al*. [30]. Therefore, it is more likely that we failed to collect eDNA in water samples. In streams, water flows continuously beside the source animals, and eDNA is unlikely to be distributed evenly in the stream. Thus, we may have failed to collect eDNA in water samples. Further studies are needed to reveal the dynamics of eDNA in flowing water.

The relationship between the abundance or biomass of tadpoles and the amount of eDNA was significantly positive over all sites combined. However, the value of *r*^2^ was not large (0.32 for abundance and 0.29 for biomass), probably because of the large difference in relationship patterns among streams. For example, in July 2017, we counted 30 tadpoles upstream, but detected only 918 copies of eDNA per second in stream C4. However, we counted 27 tadpoles and detected 48 133 copies in stream C2. Shogren *et al*. [31] showed that the transport, retention and resuspension patterns of eDNA differed according to stream substrate type, which might explain the difference in relationships between tadpole abundance and eDNA among the stream sites in this study. However, stream substrates of the survey sites were rocky with a small amount of sands and did not seem to be highly different among sites. In addition, the relationship was not constant even within the same stream: at stream C4, we counted 33 tadpoles in August 2017 and detected 10 830 copies (30 tadpoles/918 copies in July). This might also have been due to the uneven distribution of eDNA in flowing water, which can be countered by increasing the number or volume of water samples. Indeed, studies that showed significant, positive relationships sampled more water; for instance, Baldigo *et al*. [9] sampled up to 6 l of stream water and Pilliod *et al*. [13] collected three 1 l samples from each stream. Meanwhile, Spear *et al*. [14] filtered 1 l of river water (equivalent to this study) and Rice *et al*. [32] collected four 250 ml samples, and both the studies did not detect a relationship between abundance or biomass and eDNA. The methods for collecting water samples for eDNA analysis vary among studies with no fixed consensus [33]. The volume of water sample in previous studies ranged from 15 ml to more than 5 l, mostly 1–2 l [33]. As our surveys were conducted deep in the steep mountain, we could not bring more than 1 l of water from each site. In such a case, it might be better to reduce the number of sites to increase the sample volume at each survey, or to collect many subsamples of a smaller amount (e.g. 200 ml × 5 bottles). Alternatively, we could consider filtering water samples on site with single-use filters. Although we did find a positive relationship, improving sampling technique will be needed to obtain reasonable estimation power.

In conclusion, our results showed that the eDNA method can detect tadpoles in lotic systems with approximately 80% detection rate, although our sampling regime did not accurately estimate the abundance of tadpoles from eDNA quantity. Sampling a higher amount of water or a higher number of subsamples might more accurately reflect the presence and absolute amount of eDNA in water. This will increase the detection rate and decrease the false-negatives, improving the overall estimation of biomass. Further research will be needed to validate the effectiveness of these improvements.

## Ethics

The animals were handled under the permission of Kagoshima education committee no. 2015-21.

## Supplementary Material

Data_Supplementary material

## Data accessibility

The datasets supporting this article have been uploaded as the electronic supplementary material.
